# Exploring acid mine drainage treatment through adsorption: a bibliometric analysis

**DOI:** 10.1007/s11356-024-35047-2

**Published:** 2024-10-01

**Authors:** Vuyiswa Dube, Zebron Phiri, Alex Tawanda Kuvarega, Bhekie Brilliance Mamba, Lueta-Ann de Kock

**Affiliations:** Institute for Nanotechnology and Water Sustainability (iNanoWS), College of Science Engineering and Technology, University of South Africa, Florida Campus, Roodepoort, 1709 Gauteng South Africa

**Keywords:** Acid mine drainage, Adsorption, Bibliometric analysis, Bibliometrix, Biblioshiny, Heavy metals, Remediation, Schwertmannite

## Abstract

Discharge of acidic wastewater from mining activities (acid mine drainage (AMD)) is a major global environmental and public health issue. Although several approaches, including chemical precipitation and membrane technology, have been developed to treat AMD, adsorption has emerged as the most promising technology due to its cost-effectiveness and efficacy. Despite the wide adoption of adsorption in treating AMD, the evolution of research in this area remains poorly understood. To address this gap, a bibliometric analysis of the most recent literature involving the application of adsorption in AMD remediation was conducted by merging datasets of articles from Scopus (1127) and the Web of Science Core Collection (1422), over the past decade (2013–2022). This analysis revealed a yearly increase of 11% in research publications, primarily contributed by China, the United States, and South Africa. Keyword analysis revealed that natural schwertmannites and their transformations, activated carbon, zeolites, and clay minerals, are the most extensively employed adsorbents for the removal of common metals (arsenic, chromium, iron, manganese, among others). The findings underscore the need for future focuses on recovering rare earth elements, using nanoparticles and modified materials, pursuing low-cost, sustainable solutions, integrating hybrid technologies, pilot-scale studies, exploring circular economic applications of AMD sludges, and inter-continental collaborations. These insights hold significant future implications, serving as a valuable reference to stakeholders in the mining industry.

**Supplementary Information:**

The online version contains supplementary material available at 10.1007/s11356-024-35047-2.

## Introduction

Acid mine drainage (AMD), also known as acidic and metalliferous drainage or acid rock drainage (ARD), is a significant, costly global environmental and socio-economic problem that emanates particularly from the mining and mineral processing industries (Bratkova 2021). It originates from active or abandoned mines (Masindi et al. 2022) such as coal (Guo et al. 2022), gold (Tum et al. 2022), uranium (Schaeffner et al. 2015), or copper mines (Solongo et al. 2018). In addition, groundwater drills (Nishimoto et al. 2021) and urban mining of e-waste (Perera et al. 2021) have also contributed to AMD. AMD is generated through the oxidation of sulfide minerals, such as pyrites (FeS_2_), in lignite-rich rocks, mining waste, and tailings (Bratkova 2021). Historically, severe effluence has occurred in mining regions of the Appalachian Mountains (USA), Witwatersrand Basin (South Africa), Rio Tinto River (Spain), Sudbury Basin (Canada), and Shanxi and Guizhou (China), with an estimated worldwide total liability for site closure and AMD remediation of over US$100 billion (Lottermoser 2015). The estimated expenditure for the remediation of AMD at abandoned mine sites across North America was projected to be US$10 billion (Naidu et al. 2019a, b). Thus, AMD’s severity and implications require coordinated actions to tackle the problem and elevate global AMD amelioration.

AMD exposes humans and the environment to harmful and substantially elevated concentrations of dissolved metal ions (10–9000 mg/L), sulfates (16,000 mg/L), and extremely low pH levels (2–3) above the World Health Organization (WHO) provisional guideline values for portable water (Fe^2+^  < 5 mg/L, SO_4_^2−^ < 500 mg/L and pH levels (6–9)) (Kefeni and Mamba 2021). Exposure to AMD can damage the human skin, metabolism, immunity, and nervous system, culminating in conditions like skin lesions, dementia, Alzheimer’s, and Parkinsonism (Qasem et al. 2021; Singovszka et al. 2020), congenital disabilities, cancer (Chakraborty et al. 2020), and even death (Jaishankar et al. 2014). AMD also causes soil toxicity, water contamination, loss of aquatic species, siltation (Okereafor et al. 2020), and corrosion of property and infrastructure (Carnie 2022). Contamination by AMD results in water shortages and renders land areas unsuitable for habitation and agricultural purposes. Untreated AMD also results in the biomagnification, fixation, and bioaccumulation of pollutants in the food chain (Schrock et al. 2001). Therefore, remediation studies are essential for disrupting the progression of medical disorders and preserving human lives and biodiversity.

AMD treatment methods have been established to neutralize and reduce the concentration of metal ions. These include chemical precipitation (Santos Jallath et al. 2018), ion exchange (Harland 2007; Sodzidzi et al. 2024), membrane technology (Brown et al. 2015), lime neutralization (Akinwekomi et al. 2020), natural wetlands (Naidu et al. 2019a, b), and permeable reactive barriers (Kalin 2004). Nonetheless, most of these approaches are costly due to their high energy requirements, substantial need for chemical inputs, and stringent requirements for handling and disposal of toxic by-products (Naidu et al. 2019a, b). Further limitations include poor efficiencies that necessitate additional purification stages, restrictions on treating AMD due to low chemical compositions (Chen et al. 2021), and slow operating conditions (Caraballo et al. 2011).

Adsorption has shown great potential in AMD remediation due to its versatility in using a plethora of readily available materials (Goher et al. 2015), cost-effectiveness, and potential to simultaneously remove toxic metal ions at high efficiencies (> 99%) and neutralization of acidic solutions (pH levels > 6) (Kefeni and Mamba 2021). Adsorbents can be used in their natural form (De Gisi et al. 2016) or modified with other materials to produce composites through various methods (Amusat et al. 2021). Typical materials include domestic (Li et al. 2022), industrial (Yang et al. 2021), and agricultural waste products (Bacirhonde et al. 2022), clays (Gumede and Musonge 2022), zeolites (Prasad et al. 2021), nanoparticles (Chai et al. 2022; Kimpiab et al. 2022), activated carbon (Sbaffoni et al. 2018), engineered composites (Rodríguez et al. 2020), metal oxides (Rand and Ranville 2019), biochar (D. Ding et al. 2022), and alien invasive plants (Feng et al. 2021). Adsorbents have distinct features such as porosity, stability, exchange capacity, surface area, alkalinity, and sorption properties (Van Hien et al. 2020). The adsorption mechanisms are continually explored (Petronijević et al. 2022). Consequently, it has been noted that the adsorption process generates low AMD sludge volumes (Burakov et al. 2018) with beneficial properties for further applications (Spellman et al. 2022; Tum et al. 2022). Adsorption has been successfully applied for the treatment of wastewater systems (De Gisi et al. 2016), removal of dyes (dos Santos et al. 2019), treatment of brines (Paul et al. 2017), recovery of ammonium (Shakoor et al. 2021), and purification of acidic mine water (Mokgehle and Tavengwa 2021). The literature on AMD treatment through adsorption is diverse and extensive, covering various aspects of this topic. This makes it difficult to comprehensively understand the research trajectory or identify the research trends or future directions from a single perspective. Hence, a bibliometric analysis of the literature is essential for determining trends and future directions for research in this field (Visser et al. 2021).

Bibliometric analysis is a valuable tool to map the intellectual structure of a specific research field. It enables a more structured literature review, including information and detection of patterns (Ninkov et al. 2021). Web of Science Core Collection (WoSCC) and Scopus are prominent multidisciplinary databases offering comprehensive bibliographic data in various fields (Hastuti et al. 2022). WoSCC contains over 1.9 billion cited references and 85.7 million scholarly records across 254 disciplines, while Scopus boasts 87 million documents, 1.8 billion citations, and 94 thousand affiliations from 7.5 thousand publishers. Using these databases separately causes redundancy and complexity due to shared documents and fields, which requires effort to remove duplicate data. Unification of metadata is advised for effective bibliometric research, as each database has distinct characteristics (Echchakoui 2020; Kasaraneni and Rosaline 2022).

 Combining both datasets offers statistics with comprehensive time coverage, complete inclusion of new literature, and better accuracy in measuring the impact of authors (Gao et al. 2022), which improves evolutionary citations and analysis (Caputo and Kargina 2021). A few researchers have used single-based dataset analysis in wastewater treatment, such as organic adsorption (Hardyanti et al. 2023), sulfate-rich sewage (M. Ding and Zeng 2022), and mining water with heavy and precious metals (Zahoor et al. 2022). To our knowledge, this is the first bibliometric analysis using merged datasets on AMD treatment by adsorption. Other domains have reported successful bibliometric analyses using combined datasets such as sales force (Echchakoui 2020), data management (Pradhan and Zala 2021), residential social satisfaction (Biswas et al. 2021), and healthcare Internet of Things (IoT) (Ullah et al. 2022). Additionally, accessible and user-friendly methods for merging WoSCC and Scopus datasets while preserving data integrity have been proposed by utilizing R packages and Excel (Caputo and Kargina 2021; Kasaraneni and Rosaline 2022), or BibExcel and VOS viewer (Kumpulainen and Seppänen 2022). This technique provides improved convenience and a comprehensive depiction of the research landscape in the multidisciplinary field of adsorption for AMD treatment.

Bibliometric analysis of AMD treatment by adsorption has significant implications for various stakeholders. It helps understand the research dynamics, identify high-impact areas, foster collaborations, prioritize investments, and develop evidence-based policies and sustainable mining practices. This bibliometric analysis examines extensive scientific data, revealing complex evolutionary patterns in AMD remediation by adsorption. It highlights emerging trends and prospective research areas in this field. The study answers specific questions of research on AMD treatment conducted through adsorption: a) the volume and citation, b) contributions by sources, organizations, countries, and top journals, c) the nature of collaboration in adsorption in publications, d) frequent keywords, and e) research gaps and future direction.

## Bibliometric analysis

### Methods

The bibliometric analysis was performed using data from the Scopus and Web of Science Core Collection (WoSCC) databases. Several steps were followed to acquire and process the data: 1) definition of the topic of interest, 2) extraction of relevant data, 3) screening and filtering of the data, 4) exportation and merging the files, 5) application of inclusion and exclusion criteria, and 6) conduction of bibliometric analysis.

#### Data collection and analysis

**Fig. 1:**
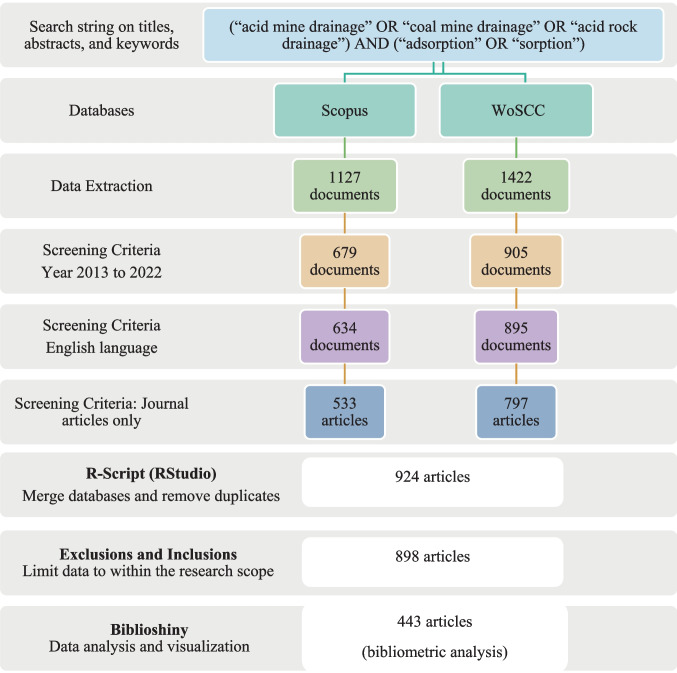
Selection and analysis of data from Scopus and Web of Science Core Collection databases

The search string (“acid mine drainage” OR “coal mine drainage” OR “acid rock drainage”) AND (“adsorption” OR “sorption”) was used to obtain the data for this study from the Scopus and Web of Science Core Collection databases. The search was restricted to English journal articles published from 2013 to 2022 and only considered the abstracts, titles, and author keywords. Only research articles were included in the search to ensure original findings. Other document types, such as conference papers, review articles, books, and book chapters, were excluded because they might have already synthesized existing knowledge rather than presenting new insights (Rigueto et al. 2022). Figure 1 summarizes the data selection procedure from each database. The search produced 1127 and 1422 publications from Scopus and WoSCC, respectively.

The search results were exported from the Scopus and Web of Science core collection databases as BibTeX and plain text files, respectively. These files contained comprehensive records of citations, bibliographies, abstracts, keywords, and cited references, among other fields. The data was merged to ensure the accuracy and reliability of a single input file for bibliometric analysis because the two databases have differences in the tag field. Echchakoui (2020) suggested that using either Scopus or Web of Science for bibliometric analysis would limit the scope and depth of the knowledge assessment. Therefore, the emerging process was executed carefully to obtain a unified dataset that would serve as a reliable input source for subsequent analyses (Fig. 1*).*

#### Merging Scopus and WoSCC datasets

The methodology employed in this study for merging the Scopus and WoSCC databases and processing the information resembles that adopted by Phiri et al. (2024). The R-script (RStudio 4.2.2 software) executes a systematic combination of multiple text files from Web of Science and bibtex files from Scopus (Fig. 1), subsequently integrating the respective datasets and concurrently eliminating duplicate entries. The detailed procedural steps are furnished in Table S1 of the Supplementary Material. After the initial data processing, Microsoft Excel commands were employed to analyze the Digital Object Identifiers (DOIs) within the resultant Excel file, thereby refining the dataset by identifying any residual duplicate records. These steps were critical for removing duplicates and irrelevant articles that were out of the scope of this research.

Therefore, this exercise resulted in an inclusive dataset focused on treating AMD through adsorption within aquatic ecosystems. It is worth noting that even after performing the necessary exclusions from 924 to 898 entries, the merged dataset still contained entries outside the study's scope. This necessitated a meticulous manual screening process, where each entry was carefully assessed through titles, abstracts, and sometimes full articles. The focus was explicitly on AMD treatment through adsorption, excluding studies on soil amendment and other unrelated topics. As a result of this rigorous screening, the number of entries was reduced from 898 to 443, enhancing the relevance of the dataset for the intended analysis (Phiri et al. 2024).

It is important to note that the breadth of research on AMD remediation through adsorption encompasses various thematic areas, including soil science studies focusing on soil additives to mitigate AMD effects. Articles deviating from the prescribed scope were excluded from subsequent bibliometric analyses to maintain focus on adsorption-based AMD treatment. These exclusions included studies on soil amendment, general heavy metal removal, and simulated aqueous solutions. Articles not directly applicable to AMD treatment or investigating the reutilization of spent adsorbents for other purposes were also excluded. The procedure underscores the importance of focusing on the specific topic of interest to avoid dilution with irrelevant studies. By excluding articles not directly related to AMD treatment through adsorption, the researchers ensured the quality and relevance of the dataset for the bibliometric analysis.

To mitigate researcher bias, two researchers conducted the screening process and criteria for inclusion/exclusion of entries by steadfastly ascribing to the research goals. The involvement of two independent researchers in this process was crucial, as it further strengthened the reliability and objectivity of the dataset. The careful manual screening process to refine the dataset for the bibliometric analysis on AMD treatment through adsorption in aquatic environments exemplifies the importance of methodological rigor and precision in research. By ensuring the dataset's alignment with the study's specific focus, the researchers could conduct a more targeted and insightful analysis of the research landscape in this domain. The information in the resulting Excel file was reviewed before conducting a bibliometric analysis. This process ensured that the data uploaded onto the platform was accurate, complete, and error-free (Caputo and Kargina 2021). The metadata extracted from the 443 documents underwent a comprehensive science mapping analysis using Bibliometrix (version 4.1.3), an R-tool developed by Aria and Cuccurullo (2017). Biblioshiny, a web-based interface designed for Bibliometrix, facilitated the generation of visualizations based on the analyzed data.

## Results and discussion

### Overview

**Fig. 2:**
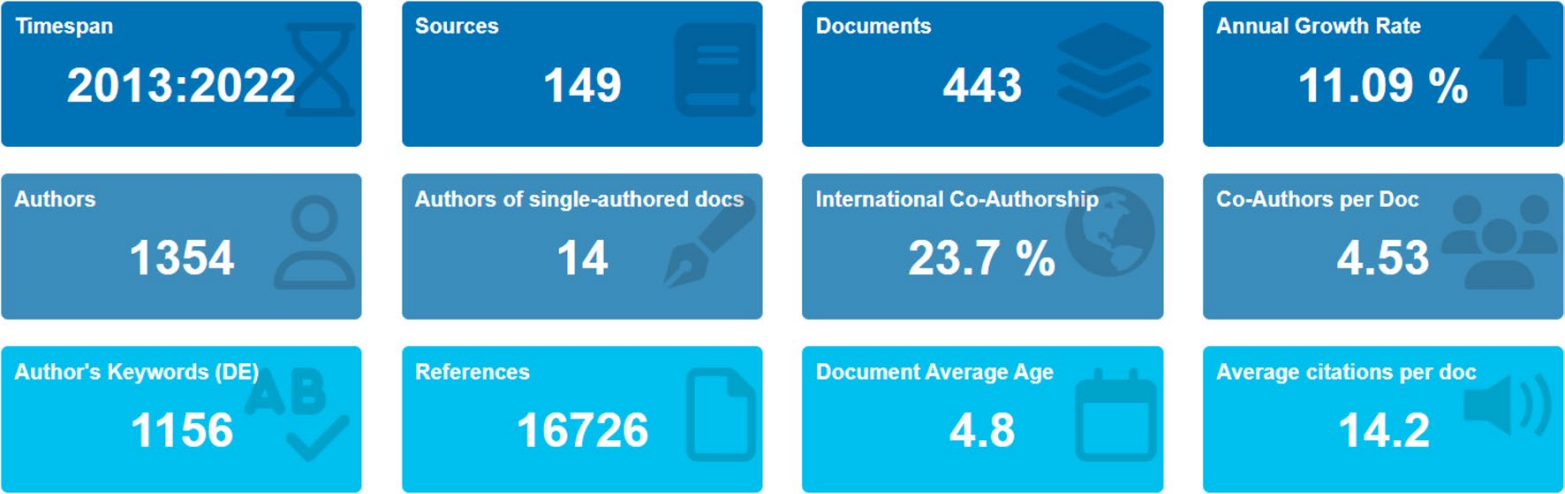
An overview of the primary information derived from the bibliometric analysis

This section presents the results of a meticulous bibliometric analysis of selected bibliometric indicators (Fig. 2). These indicators evaluate the research performance and impact of various aspects such as publication trends, citation counts, and authorship patterns. Furthermore, a science mapping technique was used with a word cloud, citation analysis, and bibliographic coupling to identify clusters and interconnections among publications. Bibliometric visualization helps classify and comprehend the research domains and trends in AMD treatment by adsorption.

This study comprehensively evaluated recent trends by analyzing a wide range of literature and existing research from various sources over the past decade. Figure 2 provides a snapshot extracted from Biblioshiny, presenting a concise overview of key insights derived from the merged database. The dataset encompasses articles with a low average document age and a substantial presence of distinctive author keywords. Noteworthy collaborative engagement among authors or institutions underscores this bibliometric analysis’s global scope and collaborative nature. These findings contribute valuable insights into the dataset's characteristics, research trends, collaboration patterns, and the article-centric nature of the study.

### Research trends and citation patterns

#### Annual scientific volume of publications and citations

**Fig. 3:**
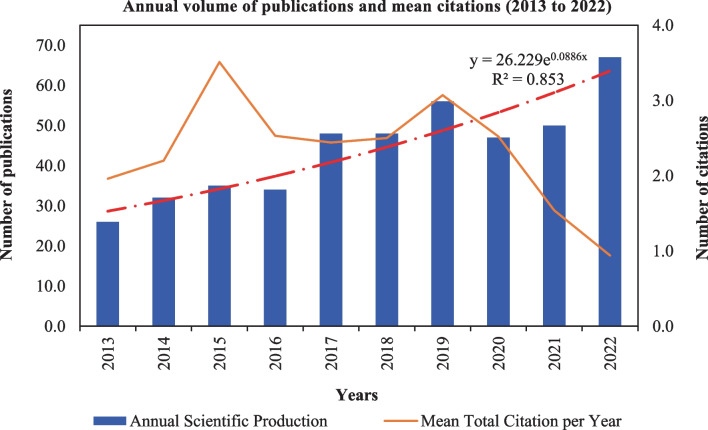
Publications and mean citation trends on adsorption for AMD treatment research

This study analyzes the publication output trends and citation patterns in adsorption and AMD treatment over the past decade (Fig. 3). Bibliometric analysis was conducted on the 443 published English-based journal articles retrieved from merging the Scopus and WoSCC datasets.

The plot shows a steady annual growth rate of approximately 11% in total outputs, from 26 publications in 2013 to 67 in 2022. Accordingly, the mean average timeline of these articles in the knowledge pool is 5 years. Interestingly, the number of research outputs peaked (67) at the end of the decade under review (2013–2022), following a sharp drop of 16% experienced between 2020 and 2021. The data was further fitted to an exponential growth curve model providing an equation of *y* = 26,229e^0.0886×^ and an *R*^2^ value of 0.82, determining the close correlation and predicting the possibility of continued activity in this research beyond 2022. In contrast, the pattern analysis shows a non-linear citation decline over time. The total citation count of the retrieved publications was 16,726 at the time of the search, with an average of 14.2 citations per publication. The corresponding annual mean total citations (Fig. 3) varied widely from 1.96 in 2013 to 0.9 in 2022. This trend had two notable peaks of 3.5 and 3.0 average citations in 2015 and 2019, respectively, a stable period of 2.5 average citations between 2016 and 2019, then a gradual decrease after that.

The number of research publications on AMD treatment has generally increased over the years, except for between 2020 and 2022, which was likely impacted by the COVID-19 pandemic, leading to limited research activities (He et al. 2023). Further data analysis suggests that research in this area will continue to increase beyond 2022. This trend can be attributed to a growing awareness of AMD’s economic, environmental, and human health effects (Cox and Moore 2022). Moreover, the Sustainable Development Goals (SDGs) aimed at achieving clean water by 2030 have garnered interest among researchers and scholars (Sadoff et al. 2020). These goals address water scarcity and pollution challenges while promoting economically viable and sustainable treatment approaches (Garrick et al. 2017). As funding opportunities in this research area are expected to increase, there will likely be a future surge in publication outputs. These findings have significant implications for future research and support in AMD treatment.

In contrast, the publication-volume trend is not aligned with the mean citation pattern. It is expected to observe a lag time between the publication of a document and its subsequent citations. The phenomenon is often referred to as citation lag (Nakamura et al. 2011). This lag occurs because newly published research takes time to be discovered, read, and cited by other researchers in their subsequent publications. Even if the number of publications in a field is increasing, contributing to a positive annual growth rate, the citations for recent articles will naturally be lower than for older publications. This occurrence is due to the time it takes for the research community to assimilate new findings and for those findings to be reflected in the citation records (Tavakolizadeh-Ravari et al. 2019). It is a normal part of the scholarly communication process, reflecting the iterative nature of research and knowledge dissemination.

Additionally, the decline in citations related to the application of adsorption for AMD remediation could be attributed to other factors. Limited access to new articles and widespread insights into the fundamentals of adsorption may have contributed to this decline over the years. Furthermore, the adsorption process may have been extensively studied and established as effective for many years, resulting in overall proven efficiency and adoption of passive systems (Webster et al. 1998; Gumede and Musonge 2022). The saturation of information among publications also results in potential redundancy and a decline in citations. (Cama et al. 2019). Alternatively, the attention of researchers may have shifted toward other treatment technologies, such as membrane technology, bioremediation, and nanotechnology, breakthroughs or innovations that may have occurred during the studied period.

### Research propagation: relevant journal sources

**Fig. 4:**
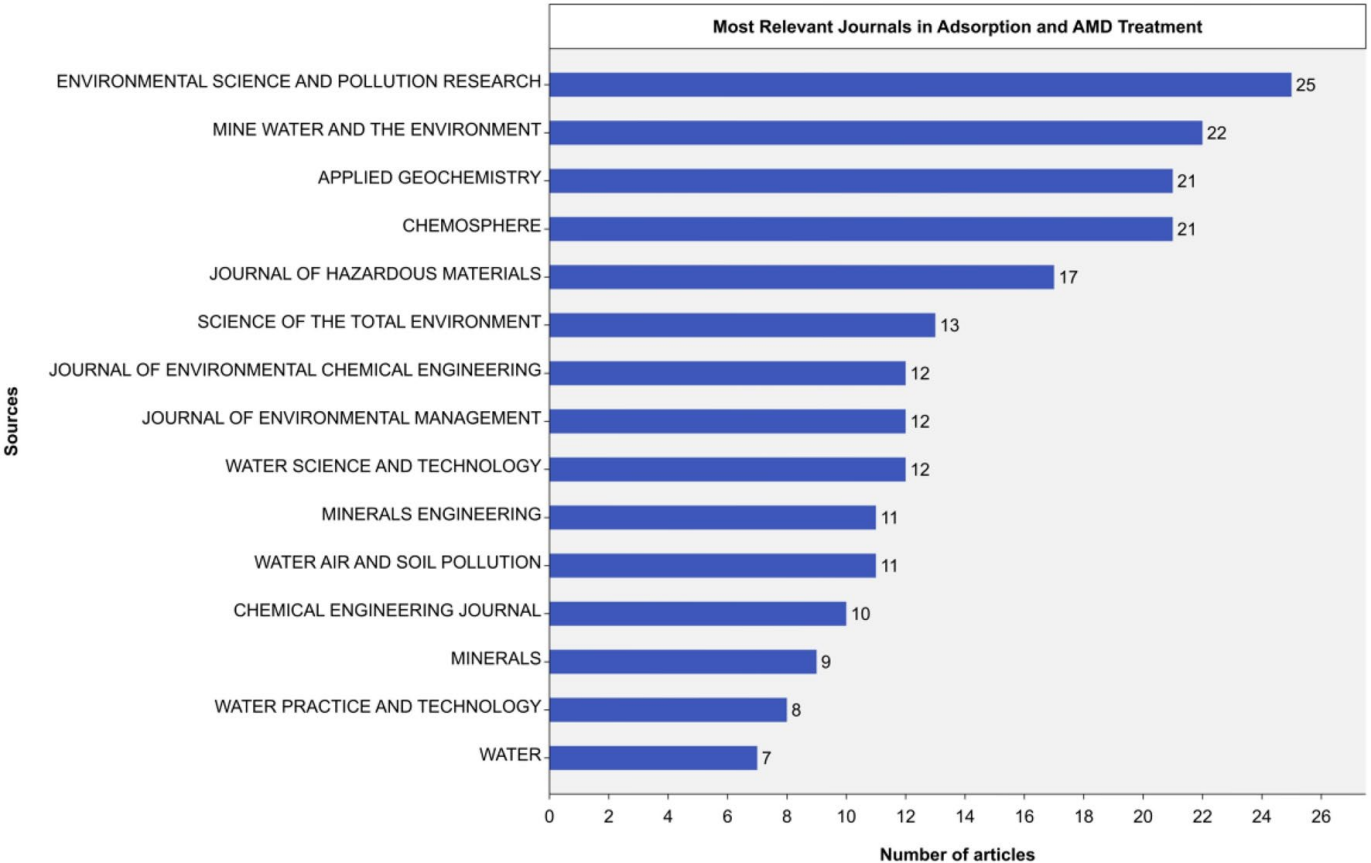
Most relevant journal sources (2013–2022)

This section analyzes the relevance of scientific outputs and impacts of various journal sources in adsorption-based AMD treatment (Fig. 4 and Table 2). The top fifteen scientifically productive journals in this field were ranked according to Bradford’s Law (Yang et al. 2016), a bibliometrics indicator used to examine 149 journal sources associated with 443 outputs on the research topic. The law works on the principle that most articles on a given topic are typically concentrated in a few core journals (Zone 1 and Zone 2), while many secondary and peripheral journals (Zone 3 and Zone 4) publish only a few articles (< 3) on the topic.

The core sources that emerged as the most popular journals included renowned contributors from Zone 1, such as “Environmental Science and Pollution Research,” “Mine Water and the Environment,” “Applied Geochemistry,” “Chemosphere,” and “Journal of Hazardous Materials.” These findings are consistent with *Bradford’s law*, which posits a decreasing exponential curve in journal productivity or relevance. Thus, a significant portion of research output concerning adsorption-based AMD treatment is concentrated within a limited number of highly productive journals.

The impact of journal sources was assessed and ranked based on various bibliometric indices, as shown in Table 1. These indices included the h-index, g-index, m-index, total citations, number of publications, and first year of publication. The analysis revealed that the field’s most productive and influential journals were *Chemosphere*, *Environmental Science and Pollution Research*, *Journal of Hazardous Materials*, *Applied Geochemistry*, and *Mine Water and the Environment*. These journals have been actively publishing articles on adsorption-based acid mine drainage (AMD) treatment research, particularly from 2013 to 2016, indicating that this is a recent and thriving area of investigation.

**Table 1:** Local impact of sources

Position	Journal Source	h_index	g_index	m_index	Total Citations	No. of Publications	Publication Year start
1	Chemosphere	12	19	1.09	391	21	2013
2	Environmental Science and Pollution Research	11	16	1.00	280	25	2013
3	Journal of Hazardous Materials	11	17	1.38	318	17	2016
4	Applied Geochemistry	10	17	0.91	302	21	2013
5	Mine Water and the Environment	10	16	0.91	280	22	2013
6	Chemical Engineering Journal	9	10	0.82	402	10	2013
7	Journal of Environmental Chemical Engineering	9	12	1.13	234	12	2016
8	Journal of Environmental Management	8	12	0.80	267	12	2014
9	Science of the Total Environment	8	13	0.80	183	13	2014
10	Geochimica et Cosmochimica Acta	6	6	0.55	230	6	2013
11	Minerals Engineering	6	11	0.60	173	11	2014
12	Water, Air and Soil Pollution	6	10	0.55	106	11	2013
13	Water Science and Technology	6	10	0.55	116	12	2013
14	Environmental Science & Technology	5	5	0.50	140	5	2014
15	Environmental Technology	4	5	0.44	91	5	2015

The h-indices for these journals ranged from 10 to 12, and their total citations ranged from 280 to 391, signifying a significant impact within the research community (Lazarides et al. 2023). Notably, the *Journal of Hazardous Materials* exhibited the highest m-index (1.38) among the top five journals, indicating a commendable balance between productivity and impact.

These findings have practical implications for researchers who want to identify the core publications in adsorption-based AMD treatment and evaluate their productivity and relevance (Naranan 1970). Furthermore, they serve as a valuable resource for researchers and scholars who want to optimize their search strategies and access the most influential sources in this field.

### Identification and evaluation of top authors

**Fig. 5:**
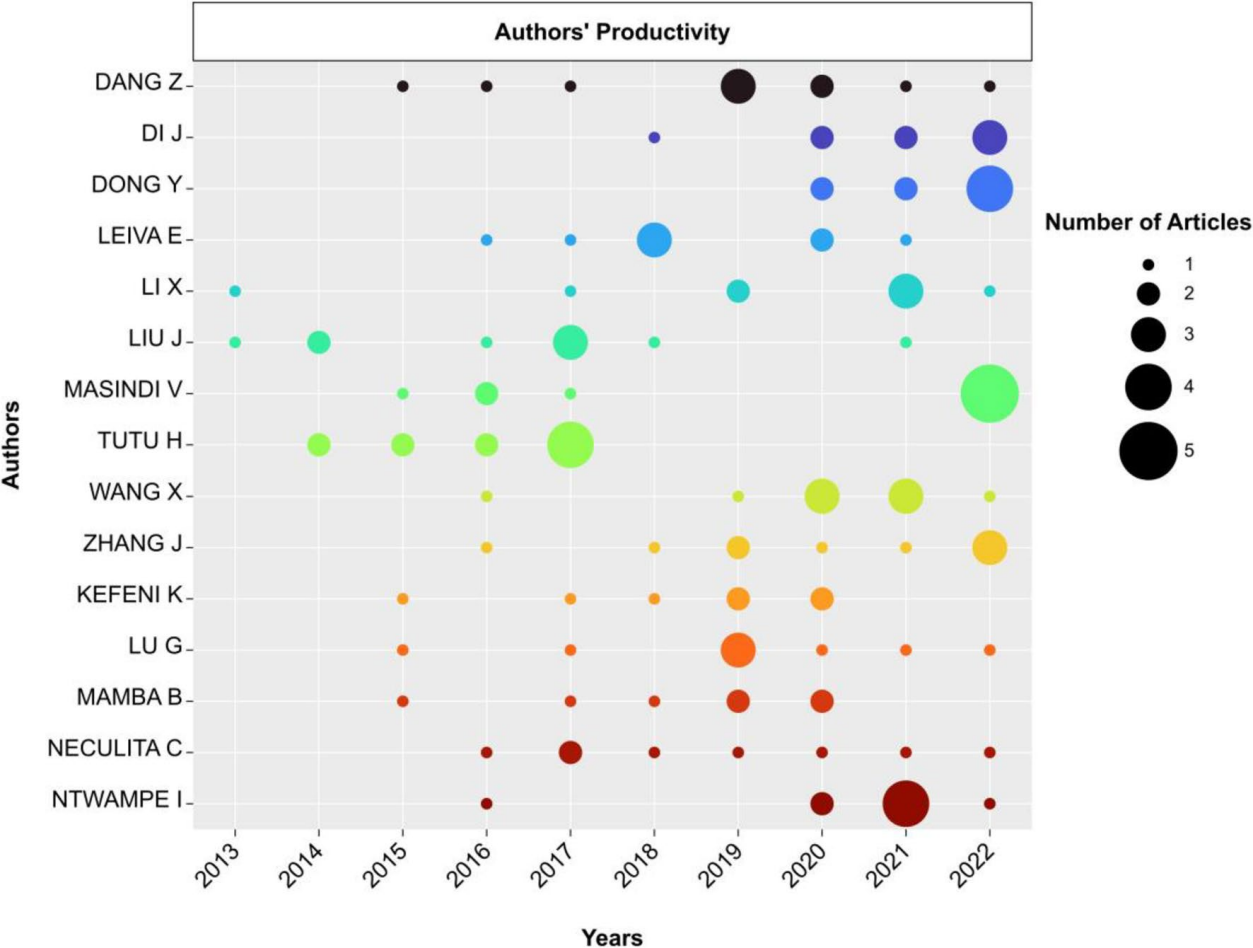
The most relevant authors in the adsorption-related AMD treatment research area

This research aims to pinpoint the foremost authors in adsorption-based AMD treatment systems by examining 1368 authors who contributed to publishing 443 articles merged from Scopus and WoSCC datasets, (Fig. 5 and Table 2)*.* The analysis showed that only 1% of the authors worked independently, indicating a high tendency towards collaboration in this research field.

**Table 2:** Local impact of authors

Item	Authors	h_index	g_index	m_index	Total Citations	No. of Publications	Publication Year start
1	Dang Zhi	9	10	1.00	251	10	2015
2	Lu Guining	8	8	0.89	196	8	2015
3	Leiva Eduardo	7	8	0.88	140	8	2016
4	Liu Jing	7	9	0.64	153	9	2013
5	Tutu Hlanganani	7	10	0.70	216	10	2014
6	Ayora Carlos	6	6	0.55	148	6	2013
7	Dong Faqing	6	6	0.55	110	6	2013
8	Guo Chuling	6	6	0.67	167	6	2015
9	Li Xuan	6	8	0.55	99	8	2013
10	Bussiere Bruno	5	6	0.45	100	6	2013
11	Fernandez-Martinez Alejandro	5	6	0.56	136	6	2015
12	Kefeni Kebede	5	7	0.56	154	7	2015
13	Mamba Bhekie	5	7	0.56	154	7	2015
14	Masindi Vhahangwele	5	9	0.56	155	9	2015
15	Msagati Titus	5	6	0,56	137	6	2015

Figure 5 shows the most prolific authors in the field of AMD treatment based on adsorption, such as *Zhi Dang, Hlanganani Tutu, Jing Liu, Vhahangwele Masindi, and Xiaoming Wang*, who published an average of nine papers each in the past decade. The seniority of these authors varied, with *Xiangdong Li* and *Jing Liu* having longer research careers in the field than *Junzhen Di* and *Yanrong Dong*, who started publishing only in the last 5 years. Most of the 15 prolific authors (approx. 70%) are likely to continue their research in this field beyond 2022. Applying Lotka’s law to all the 1156 analyzed authors (Kushairi and Ahmi 2021), it was found that 76.2% of the authors published only one paper, while the rest published multiple articles (≤ 10) during the decade. This broader perspective provides insight into the publication patterns and productivity levels across the author cohort. In general, the productivity of authors seems to improve over time. Few researchers collaborated closely (*Kebede Kefeni* and *Bhekie Mamba*, as well as *Junzhen Di* and *Yanrong Dong*), evidenced by the similarity in their respective contributions across their publications.

The findings also provide a summary of the local impact list of authors based on three critical bibliometric indicators: h-index, g-index, and m-index (Table 2). These metrics measure the impact, productivity, and influence of the author within the research field of adsorption-related AMD treatment. *Zhi Dang* emerges as the leading author on the list, with the highest h-index of 9 and a total of 251 citations, followed by *Hlanganani Tutu* (h-index: 7, total citations: 216) and *Guining Lu* (h-index: 8, total citations: 196). The other five authors on the list have similar h-index values (5), m-index (0.56), and average citations. *Fernandez-Martinez* A and *Msagati Titus* have an average citation count of 136, while *Kebede Kefeni*, *Bheki Mamba*, and *Masindi Vhahangwele* have 154 citations each. These authors have made valuable contributions to the field and deserve recognition for their impact and productivity. This information is useful for researchers and scholars as it identifies influential authors in the area and facilitates future collaborations and discussions.

### Three-field plot

**Fig. 6:**
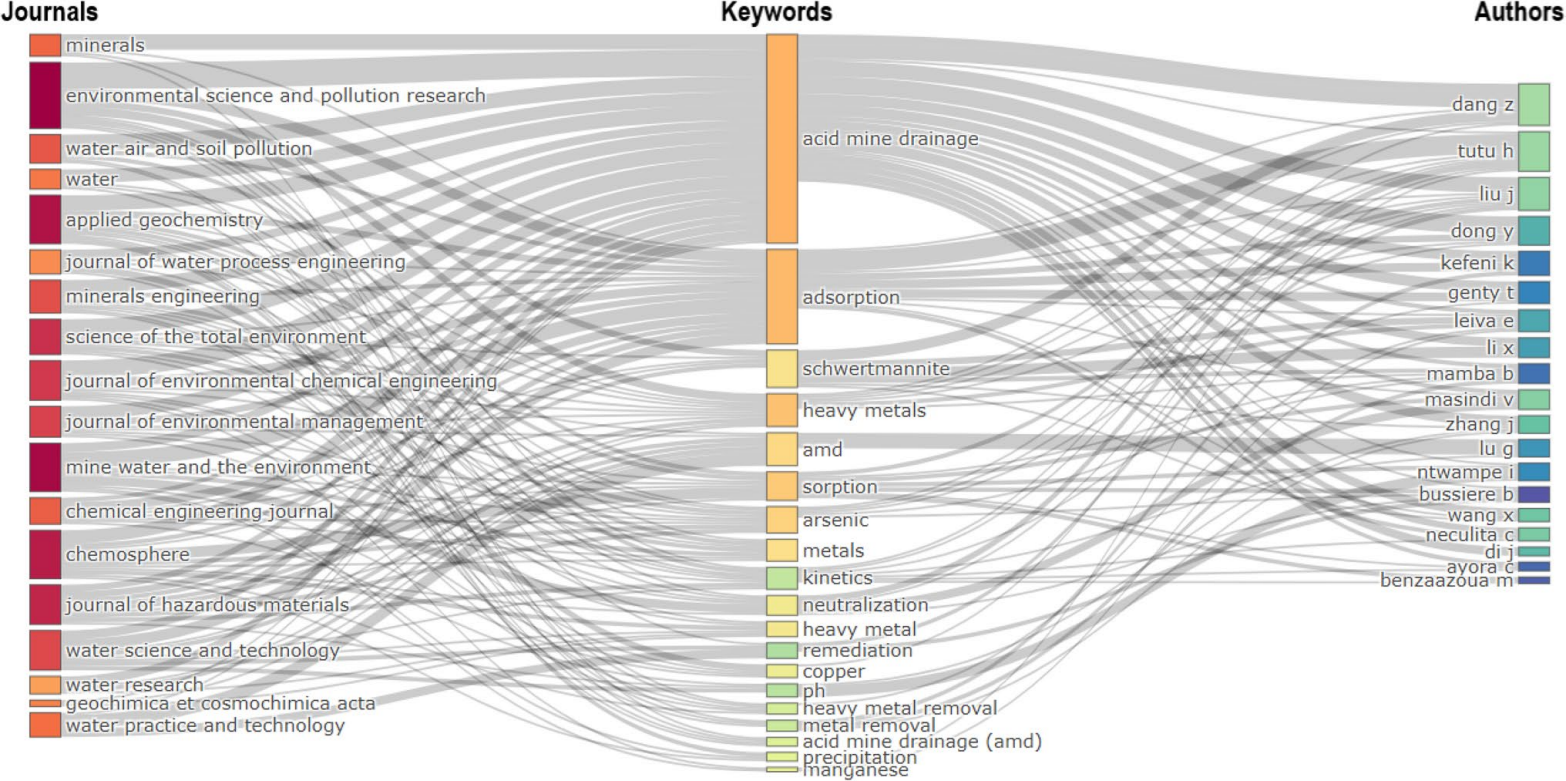
A Sankey diagram illustrating the connections between journals, keywords, and prolific authors

Figure 6 shows a three-field plot known as a tripartite Sankey diagram. This visual representation effectively outlines the interconnections among scholarly journals, key research topics derived from author keywords, and leading contributors within this domain. The core research topics highlighted include acid mine drainage, adsorption, schwertmannite, and heavy metals, all crucial for advancing environmental science and garnering significant research attention.

Schwertmannite, a nanocrystalline ferric oxyhydroxy-sulfate mineral, is generally found in acidic sulfate systems and is prevalent in environments impacted by AMD and acid-sulfate soils (Burton et al. 2009; Carrero et al. 2022; Wang et al. 2015). It has been widely recognized for its distinctive properties and is an effective adsorbent for treating AMD. Studies have demonstrated that schwertmannite can selectively adsorb significant amounts of arsenate oxyanions, phosphates, and various heavy metals commonly found in AMD (Antelo et al. 2012; Viadero et al. 2020). Schwertmannite also excels at immobilizing iron and sulfate ions, further showcasing its potential in addressing AMD and facilitating resource recovery. The specific surface area and structural features of schwertmannite significantly contribute to its effectiveness in removing contaminants from AMD (Maillot et al. 2013). Furthermore, the adsorption capacity of schwertmannite for arsenate has been a subject of interest, with studies focusing on its adsorption behavior with various oxyanions and the factors influencing its adsorption efficiency (Asta et al. 2009; Liu et al. 2015). The adsorption properties of schwertmannite have been compared with other materials like activated carbon and zeolites, showing favorable results and highlighting its efficacy in AMD treatment (Shu et al. 2019; Jin et al. 2021).

Moreover, the utilization of schwertmannite in AMD treatment has been explored in various contexts, such as its transformation under different conditions and its interactions with other compounds like Fe(II) and Cu(II) (Carrero et al. 2016; Yang et al. 2020). Studies have investigated the impact of factors like sulfate availability on the stability of schwertmannite, highlighting the importance of sulfate concentrations in influencing its transformation and persistence (Burton et al. 2013). Additionally, research has delved into the thermal transformation of schwertmannite, demonstrating how high temperatures can lead to its conversion to other minerals, affecting the mobility of metal(oid)s (Mulopo 2022).

Recent studies have explored an innovative approach to AMD treatment by synthesizing schwertmannite using composite oxidants like H_2_O_2_ and Na_2_O_2_. The process removes heavy metal ions and sulfate ions from AMD and increases the water pH, thereby reducing its acidity (Chen et al. 2023). Moreover, the synthesized schwertmannite can be reused as an adsorbent for heavy metal ions, contributing to sustainable AMD management (He et al. 2024).

The treatment mechanism follows a systematic two-step approach. First, schwertmannite is produced to remove contaminants, and then it is separated from the solution. In the subsequent step, additional oxidants are introduced to eliminate residual ions and neutralize acidity (Chen et al. 2023). This innovative method harnesses the resource potential of AMD by utilizing contaminants like Fe ions and SO_4_^2−^ to form schwertmannite (Chen et al. 2023; He et al. 2024).

The plot in Fig. 6 offers an overview of the distribution of research topics among different authors and the journals they choose to publish. It provides invaluable insights into the trends and patterns of research in this field, serving as a guidepost for future research efforts and collaborations. These findings hold the potential to drive advancements in the field and the development of sustainable solutions to address environmental challenges, inspiring the academic community to delve deeper into these areas.

### Global research productivity: leading institutions

**Fig. 7:**
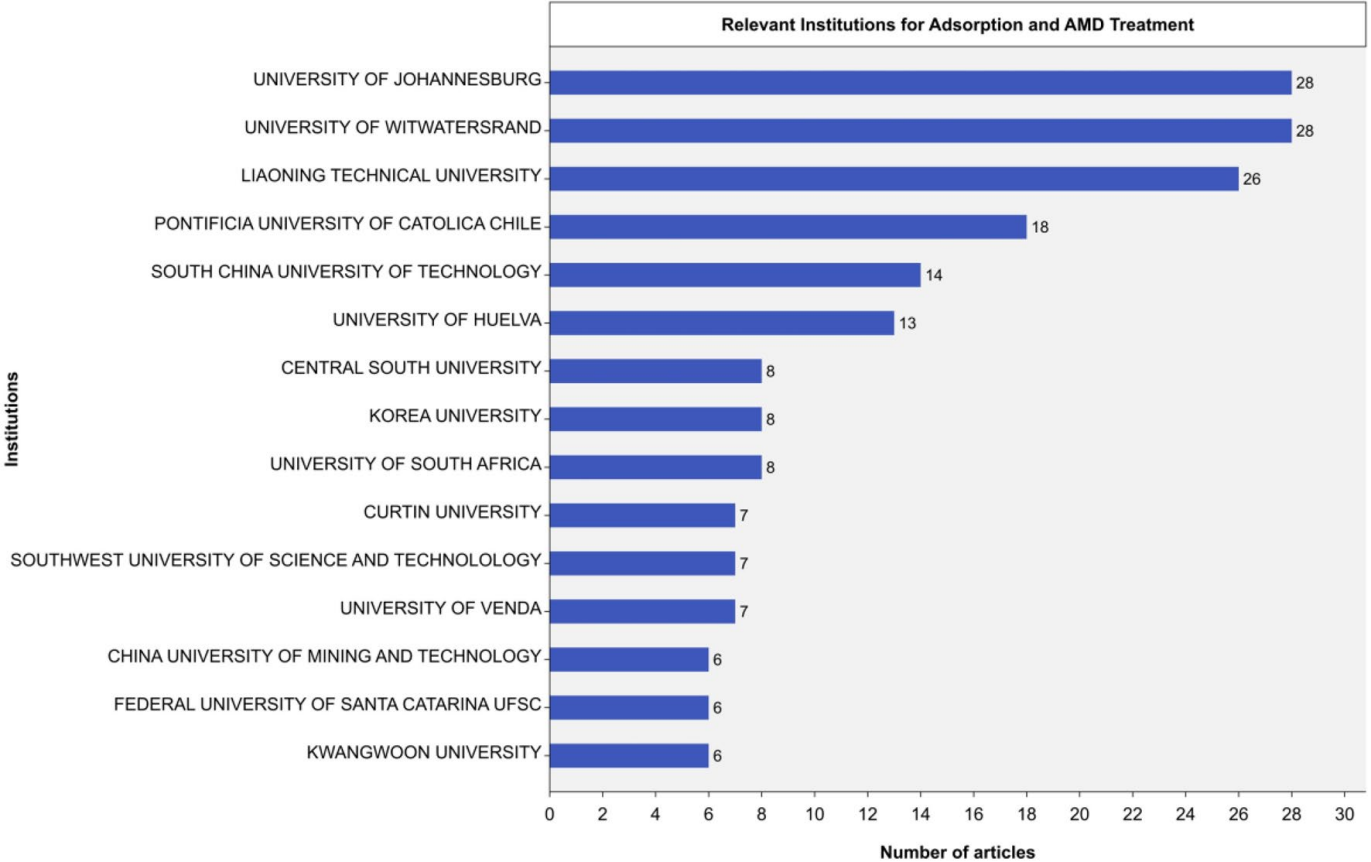
The leading institutional affiliations (2022)

This section shows the global research output of the 626 academic institutions that published 443 journal articles on adsorption-based AMD remediation. Figure 7 ranks the top 15 institutions based on their contribution to this field. The top-ranked institutions have diverse research interests, from specific topics to interdisciplinary approaches.

The results showed that the *University of Johannesburg* and the *University of the Witwatersrand* are the leading institutions in this field, with 28 research outputs each as of 2022. These institutions have shown a consistent and growing publication output since 2014. *Lianoning Technical University* ranked second with 26 publications, followed by the *Pontificia Universidad Católica de Chile* and the *South China University of Technology* with 19 and 14 publications, respectively. Notably, the *Pontificia Universidad Católica de Chile* started publishing on this subject in 2016 and has increased its output since 2018. Similarly, the *South China University of Technology* and *Lianoning Technical University* have shown an upward trend in their publication output over the years (2013–2022).

This investigation reveals a noteworthy trend of the emerging prominence of institutions in Southern Africa and Asia, indicating their increasing significance as contributors to research in the field. This growth may result from the private sector or government-led initiatives that provide funding for research endeavors and the growing prevalence of international collaborations between institutions to advance scientific knowledge.

The number of articles published determines the research output of each institution in this study. However, a more comprehensive assessment of research productivity could use other metrics, such as citations and impact factors. The study does not identify specific factors that explain the success of these institutions in adsorption-based AMD remediation research. However, their achievements may have resulted from many factors, including robust research infrastructure, funding, the urgency to address the AMD problem, scarcity and provision of clean water, and collaboration with industry partners. These institutions are distributed across different regions worldwide, which suggests that research productivity is broad and open to institutions in developed countries. Nonetheless, this study offers valuable insights into the current research landscape and the global contributions of academic institutions.

### Country scientific production: an analysis of author affiliations

**Fig. 8:**
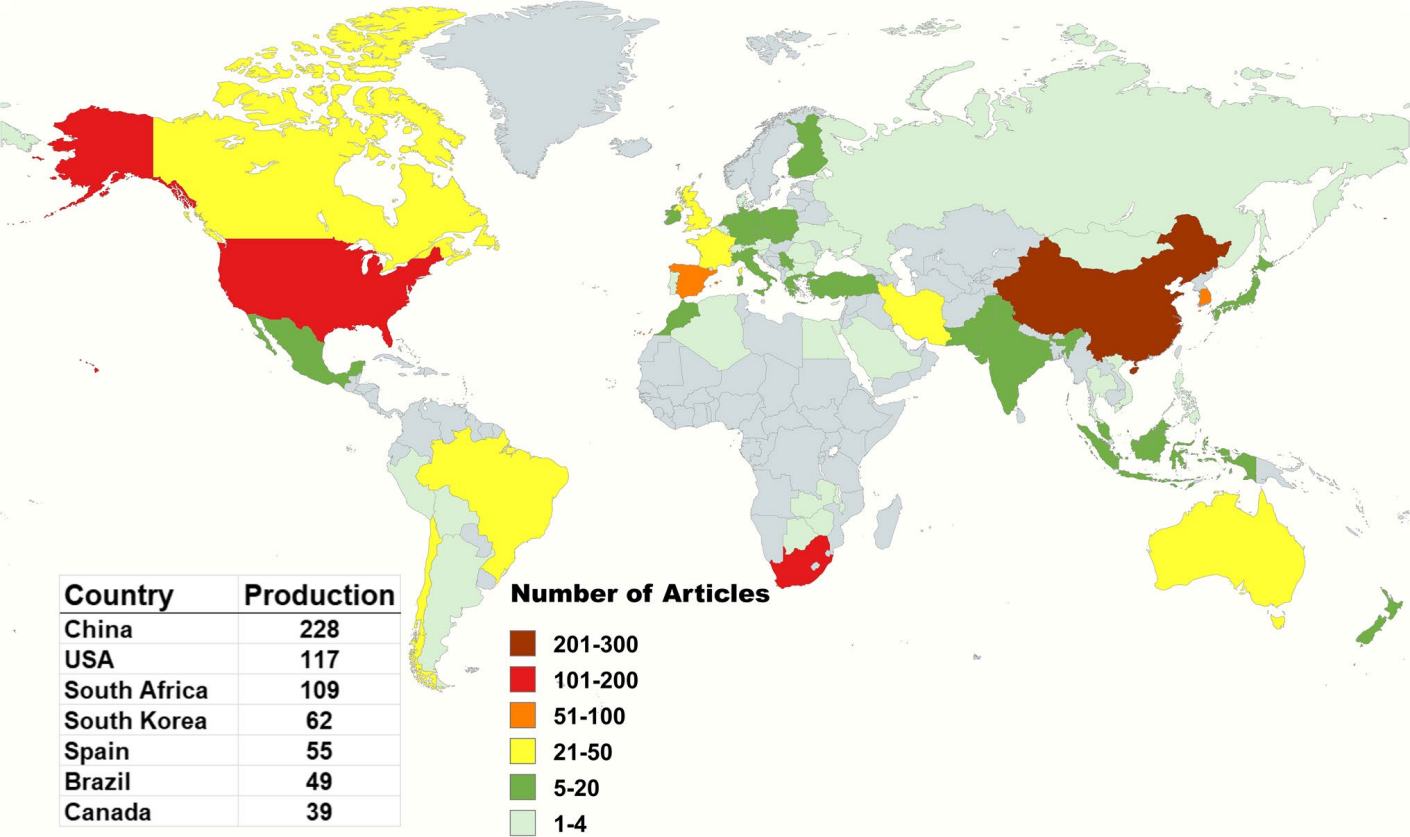
Scientific productivity of countries (created with mapchart.net)

To identify the countries that have contributed the most to the research on the application of adsorption for AMD treatment, a bibliometric analysis was conducted on the affiliations of the authors of the publications within 149 nations around the globe (Fig. 8). Bibliometrics counts the number of “author appearances by country affiliates” for each article (Pritchard 1969). Each country gets one point if an article has authors from different countries (Phiri et al. 2024). This method helps track research output and impact and better understand the global scientific landscape. Data was extracted from Biblioshiny and visualized using a Map Chart.

China had the highest research outputs, with 228 publications, followed by the United States of America (117) and South Africa (109). The prevalence of knowledge base dominance in these countries is evident through the long mining history, and they are home to some of the world's largest mining operations. Consequently, these nations benefit from supportive government funding for research and development initiatives, which allows the creation of well-established affiliations and institutes with dedicated facilities (Seoh and Im 2020).

These countries also adhere to a regulatory framework committed to driving research toward sustainable development and environmental protection through effective treatment methods for AMD. It is noteworthy that there is a relative lack of attention given to AMD pollution in Africa compared to other regions, despite the severe water scarcity and chronic human health challenges faced by this continent (Dagestani et al. 2022; Feris and Kotze 2017). This study highlights the need for research attention and funding between developed and developing nations, promoting the possibility of collaborative work between countries.

Understanding the intricate and diverse characteristics of AMD, which vary significantly from region to region due to the complex interplay of mining legacies, local geology, mining practices, and climate conditions (Ly et al. 2019; Ochieng et al. 2010), is crucial. The varying concentrations of contaminants, pH levels, and types of metals in AMD underscore the need for tailored adsorption approaches (Skousen et al. 2016). These variations can influence the selection and efficiency of AMD remediation methods, including adsorption techniques (Zhan et al. 2019). Given the multifaceted nature of AMD, it is essential to emphasize that more than a single approach to adsorption techniques is needed.

In addition, it is important to note that economic and technological factors have a global reach and play a significant role in choosing adsorption techniques (Rashid et al. 2021). Developed countries may have access to more advanced and costly adsorbents while developing countries might rely on more cost-effective, locally available materials. This disparity in resources can significantly impact the approach to AMD remediation worldwide. Furthermore, environmental regulations and sustainability goals are key drivers in the choice of adsorption technology (Busetty 2019). Some countries might prioritize using environmentally friendly adsorbents, such as those derived from agricultural waste, over more traditional activated carbon. This shift in focus towards sustainable solutions is a significant development in AMD remediation.

However, this study did not conduct a country-specific analysis to assess the potential applications of adsorbents. A study by Phiri et al. (2024) revealed that sewage sludge and rice straw are the predominant waste biomasses utilized for biochar production to treat heavy metal contamination in aqueous environments. While the fundamental principles of adsorption remain the same, the practical application of this technique for AMD remediation can vary between countries based on local AMD characteristics, economic considerations, technological capabilities, and environmental policies. Remediation strategies must be adaptable to these local conditions to ensure effective treatment of AMD.

### Word cloud analysis

**Fig. 9:**
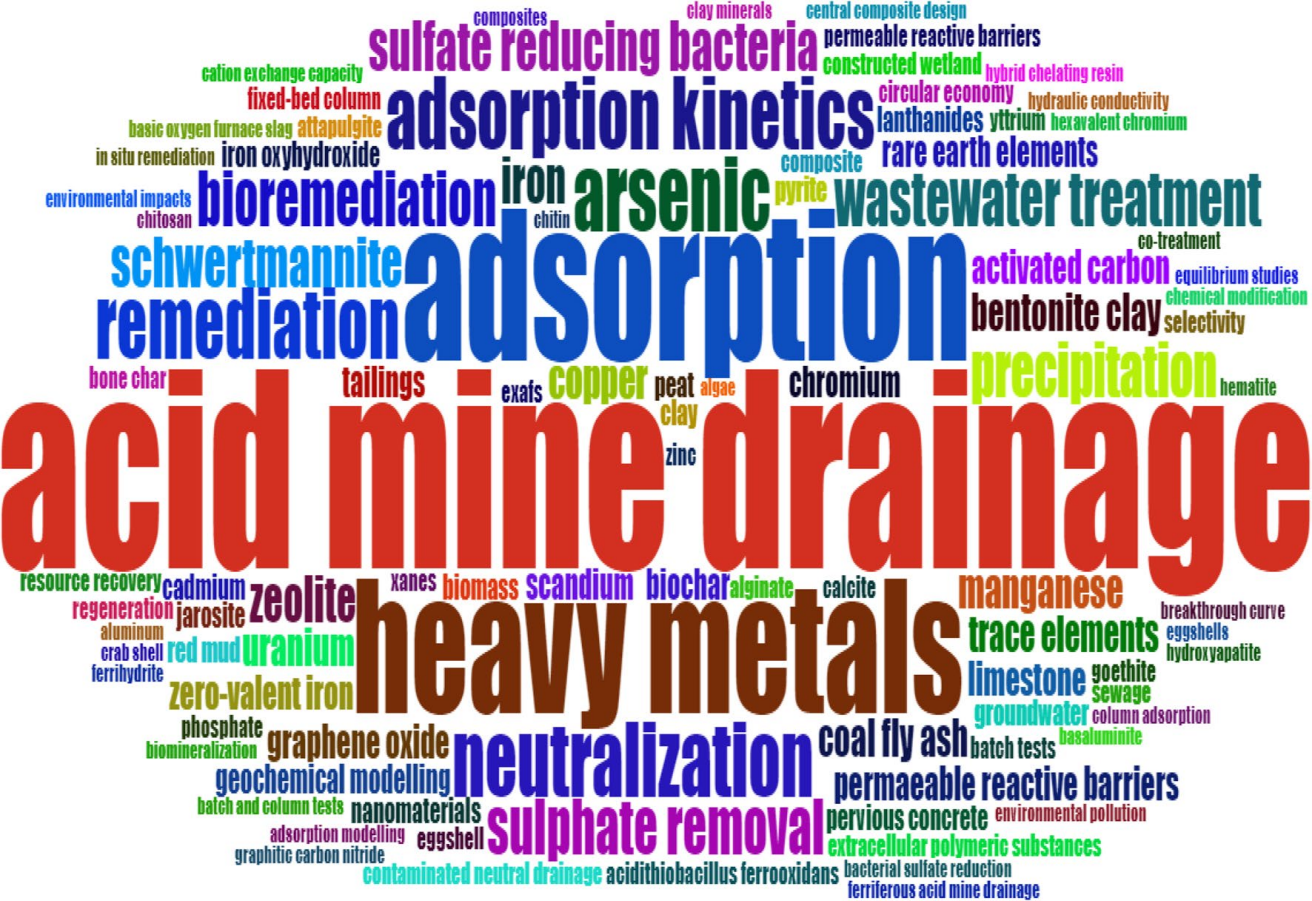
Word cloud for occurrence of 100 authors’ keywords

This study analyzed the evolution of the authors’ keywords related to adsorption and AMD treatment over 2013–2022. As a bibliometric indicator, Word Cloud analysis assessed the frequency of keywords extracted from 443 articles from the merged WoSCC and Scopus datasets. The larger the font size of the words, the more frequently they appear in this dataset. Synonymous words were combined into one term for a more accurate thematic analysis. The data of the top 100 keywords were extracted and visualized (Fig. 9) through the Biblioshiny, a web-based application.

The analysis of the word cloud based on the initial search keywords “acid mine drainage” and “adsorption” revealed the prominence of terms like “heavy metals” and “arsenic,” which emerged as prominent pollutants. At the same time, “schwertmannite” stood out as the most utilized adsorbent. These terms indicate the focus on pollutants and adsorbents in the literature related to AMD remediation. While adsorption, mainly using schwertmannite, is a significant method for AMD treatment, the analysis also highlighted the utilization of other techniques, such as neutralization, precipitation, and bioremediation involving sulfate-reducing bacteria (Dovorogwa and Harding 2022; Mosai et al. 2024), acidithiobacillus ferrooxidans (Song et al. 2022), and algae (Ramasamy et al. 2019).

Researchers have expanded their perspective on AMD, recognizing it as wastewater and a potential source of rare earth elements (Xia et al. 2023). Studies have shown that AMD can be a resource for rare earth elements, indicating a shift towards viewing such polluted waters as opportunities for resource recovery. This shift in mindset is crucial for sustainable environmental management practices. The word cloud analysis provides insights into the multidimensional approach toward AMD management, encompassing various remediation techniques and recognizing the potential of AMD as a source of rare earth elements. This holistic view underscores the importance of integrating environmental remediation with resource recovery strategies for sustainable mining practices.

### Conceptual structure: keyword co-occurrence network

**Fig. 10:**
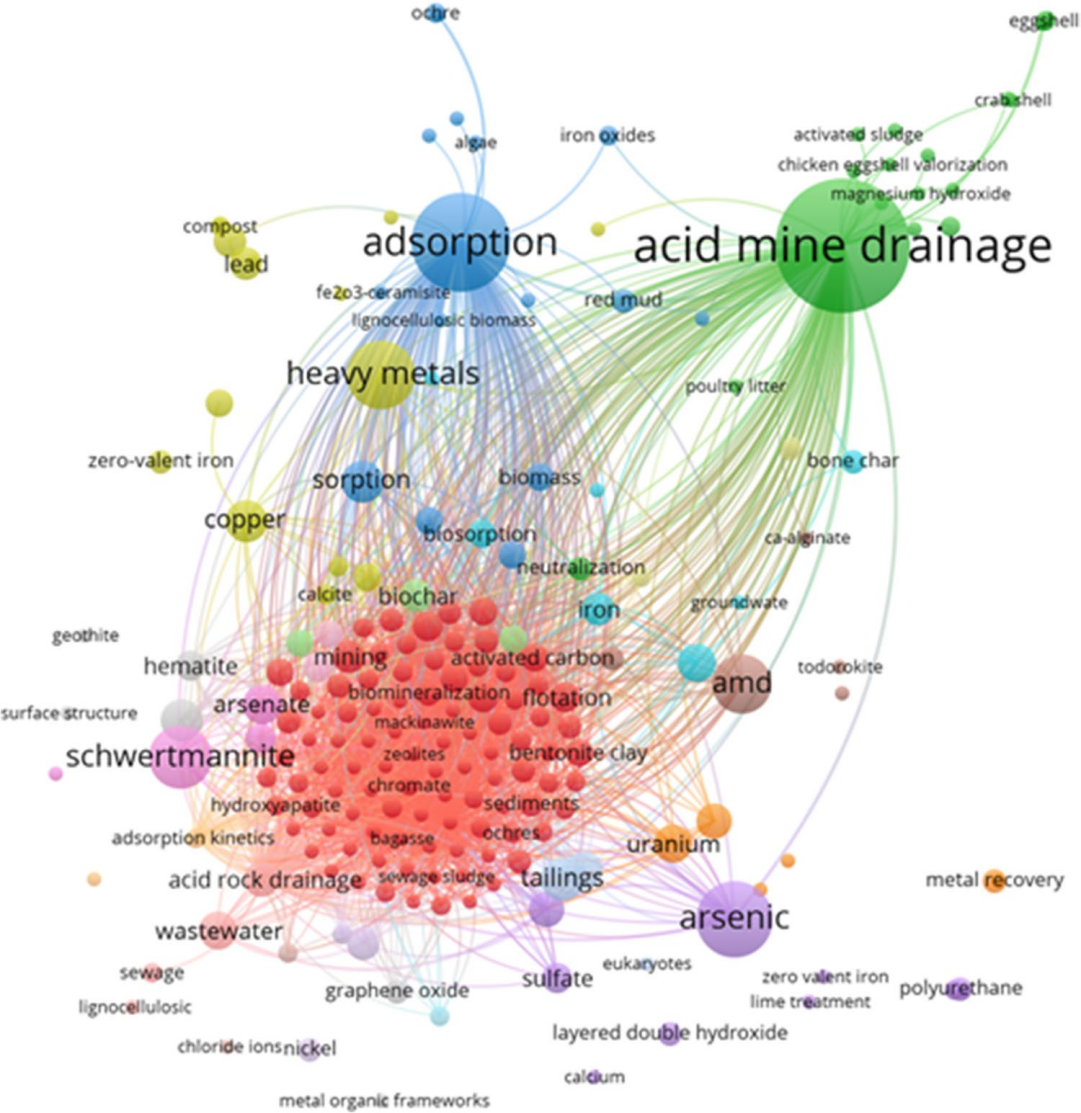
Keyword co-occurrence network, generated by VOSviewer (version 1.6.19)

The study used a conceptual co-occurrence network to analyze the relationships between indexed keywords to understand the research progress and interests related to adsorption-based AMD remediation (Table 3 and Fig. 10). The network was based on a bibliometric analysis of 1156 keywords associated with the 443 articles merged from Scopus and WoSCC related to adsorption-based AMD treatment. The distribution of occurrences was 79% (1190 keywords with 1 occurrence), 15% (231 keywords, 2 occurrences), and only 1% (15 keywords, > 10 occurrences). The top 20 keywords with the most significant link to this research area are given in Table 3. Based on this information, the co-occurrence network was then designed and visualized through the VosViewer by considering a minimum number of occurrences (2) of keywords and calculating the link strength between words. The data was verified to exclude irrelevant words, resulting in 247 keywords, shown by different-sized and colored spherical nodes and interconnecting links (Fig. 10). The size of the spheres or nodes represents the prominence or significance of a particular keyword within the network context, often determined by the frequency or occurrence count. At the same time, the thickness of the line is directly proportional to the level or strength of the co-linkage of keywords.

**Table 3:** Co-occurrence indexed keywords and link strengths

Ranking	Keyword	Occurrence	Total link strength
1	acid mine drainage	121	970
2	adsorption	62	856
3	arsenic	36	777
4	heavy metals	29	757
5	schwertmannite	24	739
6	AMD	21	752
7	metals	14	713
8	pH	12	718
9	copper	10	705
10	tailings	11	705
11	pyrite	10	701
12	sorption	10	700
13	manganese	8	696
14	wastewater	8	695
15	antimony	7	693
16	bioremediation	8	692
17	iron	6	692
18	uranium	8	692
19	biochar	6	691
20	rare earth elements	7	689

The present study utilized network analysis to investigate the interconnections among 247 nodes or concepts, which were categorized into 22 clusters (Fig. 10). Notably, 23% of these clusters exhibited a distinct nature, each comprising more than 10 concepts. Remarkably, most of the network demonstrated connectivity to at least one concept, indicating a cohesive structure.

The most prominent cluster comprises 117 items and primarily represents various adsorbents, denoted by red-colored nodes on the map. This cluster encompasses the adsorbents’ performance in terms of kinetics and mechanisms and their application techniques, such as flotation, flocculation, and modifications through biomineralization. Additionally, optimization tools such as PH (pH), RE (redox), EQ (equilibrium), C (program written in C), and PHREEQ also feature in this cluster.

The second most prominent cluster (green nodes) has 24 concepts centered around “adsorption,” with the highest occurrence of 62, 168 total link strength, and 68 links. Other keywords (with their respective occurrences, total link strengths, and links) in this cluster include “sorption” (10, 180, 171), “biomass” (4, 173, 170), and “carbonization” (4, 174, 169). On the other hand, the third cluster (denoted by dark blue nodes) has 21 concepts encompassing various adsorbents associated with AMD remediation. These include composites, nanoparticles, and microbial-based adsorbents. This cluster’s prominent topics are AMD neutralization and sludge modification byproducts obtained during lime treatment. The top node is “acid mine drainage” (121, 271, 90), followed by “manganese” (8, 180, 170), “neutralization,” “co-treatment,” and “bentonite,” all with the highest occurrence of 3, total link strength of 170, and 169 links.

The yellow nodes represent the fourth cluster, consisting of 13 items. The primary nodes in this cluster are “arsenic” (36, 210, 175), “antimony” (7, 183, and 169), “chromium” (5, 175, and 169), and “sulfates” (5, 171, and 170). The fifth cluster, denoted by purple nodes, has 11 items. The main nodal points are that of “heavy metals” (29, 197, and 175), “pH” (12, 186, and 169), “iron” (6, 177, and 169), “biosorption” (4, 174, and 170), and “molybdenum” (2, 169, and 169). Schwertmannite, pyrites, arsenates, and biochar often link to cadmium, chromium, and phosphates as pollutants. The sixth cluster’s main components, denoted by light blue nodes, have nine items with notable frequencies, connections, and intensities.

The drawbacks of word clouds, which do not display the complex and rich relationships among keywords, can be overcome through keyword co-occurrence network visualization. This method enables the connections and contexts of different concepts to be seen and understood clearly and informatively (Radhakrishnan et al. 2017). As depicted in Fig. 10, the keyword co-occurrence network analysis provides valuable insights into the interconnectedness of terms within a specific research domain. While the appearance of “acid mine drainage” and “adsorption” can be attributed to their use as search terms, the prominence of terms such as “schwertmannite,” “heavy metals,” and “arsenic” in the network suggests their significance within the context of the analyzed literature.

Schwertmannite, a mineral commonly associated with acidic mine environments, is known for controlling the mobility of heavy metals such as arsenic (Zhang et al. 2018). The presence of these terms in close proximity within the network indicates a strong thematic relationship, highlighting the importance of schwertmannite in heavy metal immobilization in acid mine drainage settings. The association of “metal recovery” with “rare earth elements” and “tailings” in the keyword co-occurrence network suggests a focus on the recovery of valuable metals, particularly rare earth elements, from mining waste materials such as tailings. This connection underscores the growing interest in sustainable mining practices and the potential for resource recovery from secondary sources.

As indicated by various colors, the clustering of keywords in the network likely represents distinct thematic groupings or research subfields within the broader mining and environmental science domain. These clusters may reflect common research trends, emerging topics, or interconnected concepts within literature. The keyword co-occurrence network analysis offers a systematic approach to identifying key terms, relationships, and thematic patterns within the body of literature. By leveraging network analysis techniques, researchers can uncover hidden connections, prioritize research areas, and gain deeper insights into the underlying knowledge structure in a specific field.

### Social structure: global collaboration network

**Fig. 11:**
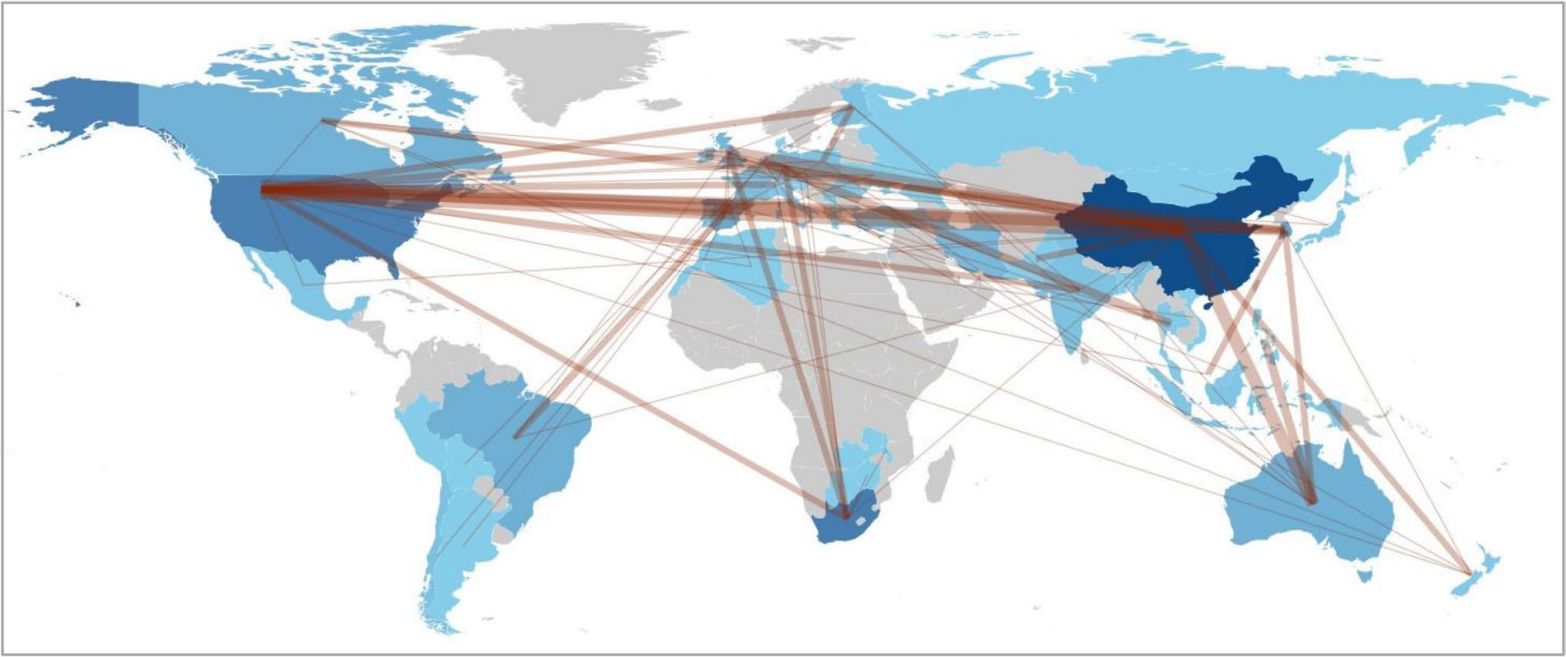
Global collaboration network

This study investigates the international collaborations and co-authorship patterns among researchers who publish articles on AMD remediation using adsorption (Fig. 11). The data was obtained from a bibliometric analysis of the social structure of 443 articles, and the collaboration network of 37 countries was visualized through Biblioshiny web-based application. The network parameters were defined for a spherical layout and a walktrap clustering algorithm and normalized by association.

The network encompasses 37 countries, indicating substantial global interconnectedness among researchers. Notably, China, the United States, and South Africa emerge as the foremost productive nations, albeit with contrasting collaboration networks. China and South Africa display larger networks, implying extensive external collaborations, whereas the United States exhibits a more insular approach with a smaller network. This suggests a higher inclination towards internal collaborations within the United States. Furthermore, the co-authorship patterns in the network reveal the influence of geographical proximity and socio-economic similarity on collaborations, such as Zimbabwe, Malawi, and South Africa. However, unique collaborations transcend continental boundaries and socio-economic disparities, for instance, New Zealand and USA, Korea and Canada, Serbia and Japan, Turkey and Iran, or Brazil and India, all reflecting a shared dedication to surmounting cultural and economic obstacles in addressing AMD challenges (Mohamed et al. 2022).

The findings presented in this analysis provide significant contributions to understanding the landscape of international collaboration within the realm of AMD remediation through adsorption techniques. The study underscores the crucial role of collaborative research in driving progress in this field and emphasizes the profound value of international cooperation in addressing the challenges associated with AMD. These insights highlight the need for continued efforts to foster global partnerships and knowledge exchange to tackle this issue and promote advancements in AMD remediation effectively.

### Intellectual structure: trend topics

**Fig. 12:**
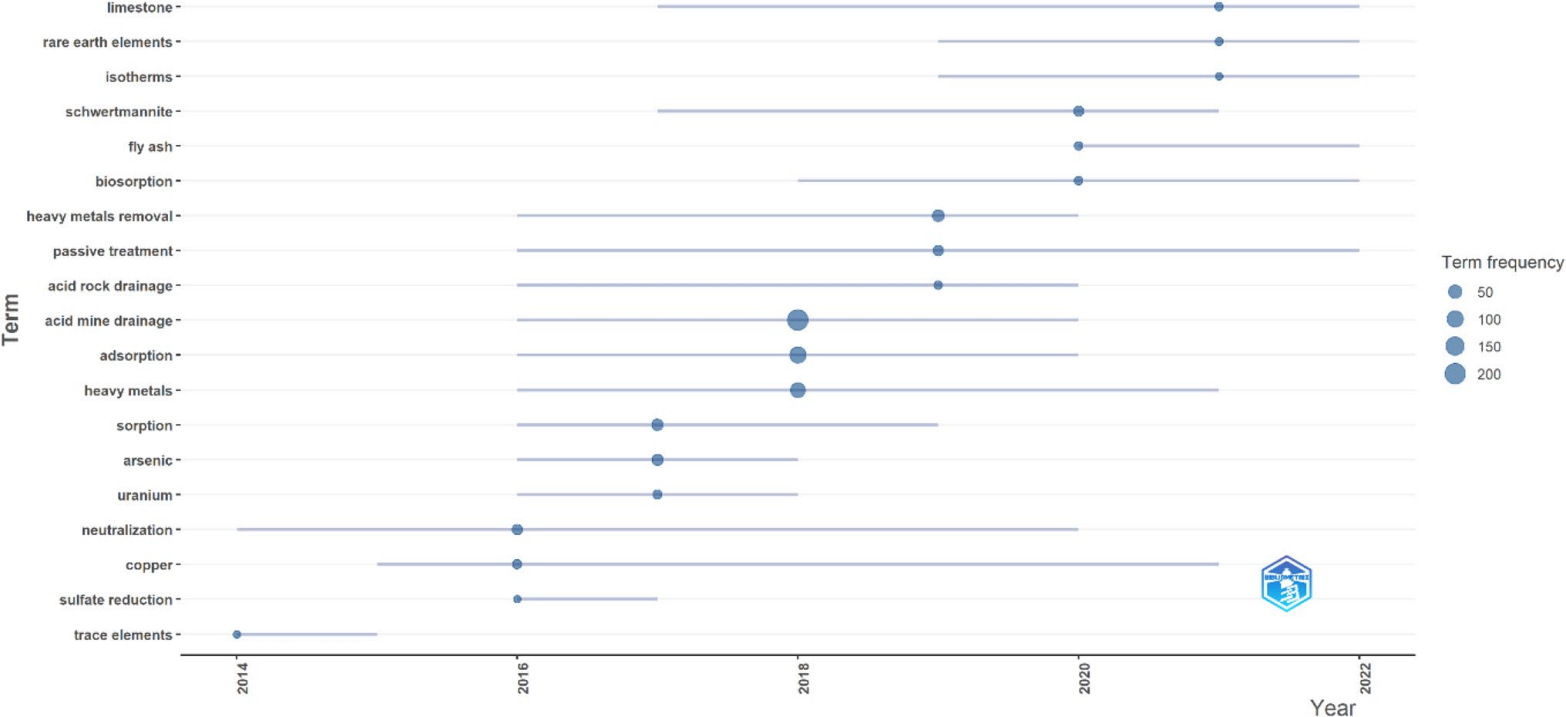
Evolution of trending topics associated with adsorption-based AMD treatment (2013–2022)

Analyzing trends in the frequency of authors’ keywords over time through bibliometric analysis provides valuable insights into the evolution of research hotspots. A minimum term frequency of 2 and an annual appearance below 7 was used. Before the analysis, an embedded algorithm within the application consolidated synonymous terms. Figure 12 presents a snippet from Biblioshiny that illustrates the trending topics and their corresponding frequencies spanning from 2013 to 2022. A shift in focus from trace elements to heavy metals and rare earth elements has been observed in adsorption-based AMD remediation. This evolution signifies a growing interest in addressing toxic heavy metals and resource recovery in AMD treatment strategies. Additionally, the consistent study of AMD neutralization and the increasing traction of passive treatment techniques highlight the ongoing efforts to develop sustainable and effective remediation methods (RoyChowdhury et al. 2015).

By tracking changes in keyword frequency, researchers can summarize research trends and predict future directions in AMD remediation. Such analytical approaches help understand the dynamics of research areas and guide future investigations toward effectively addressing emerging challenges. This systematic approach allows for a comprehensive assessment of research progress and helps identify gaps that require attention to advance remediation technologies.

**Fig. 13:**
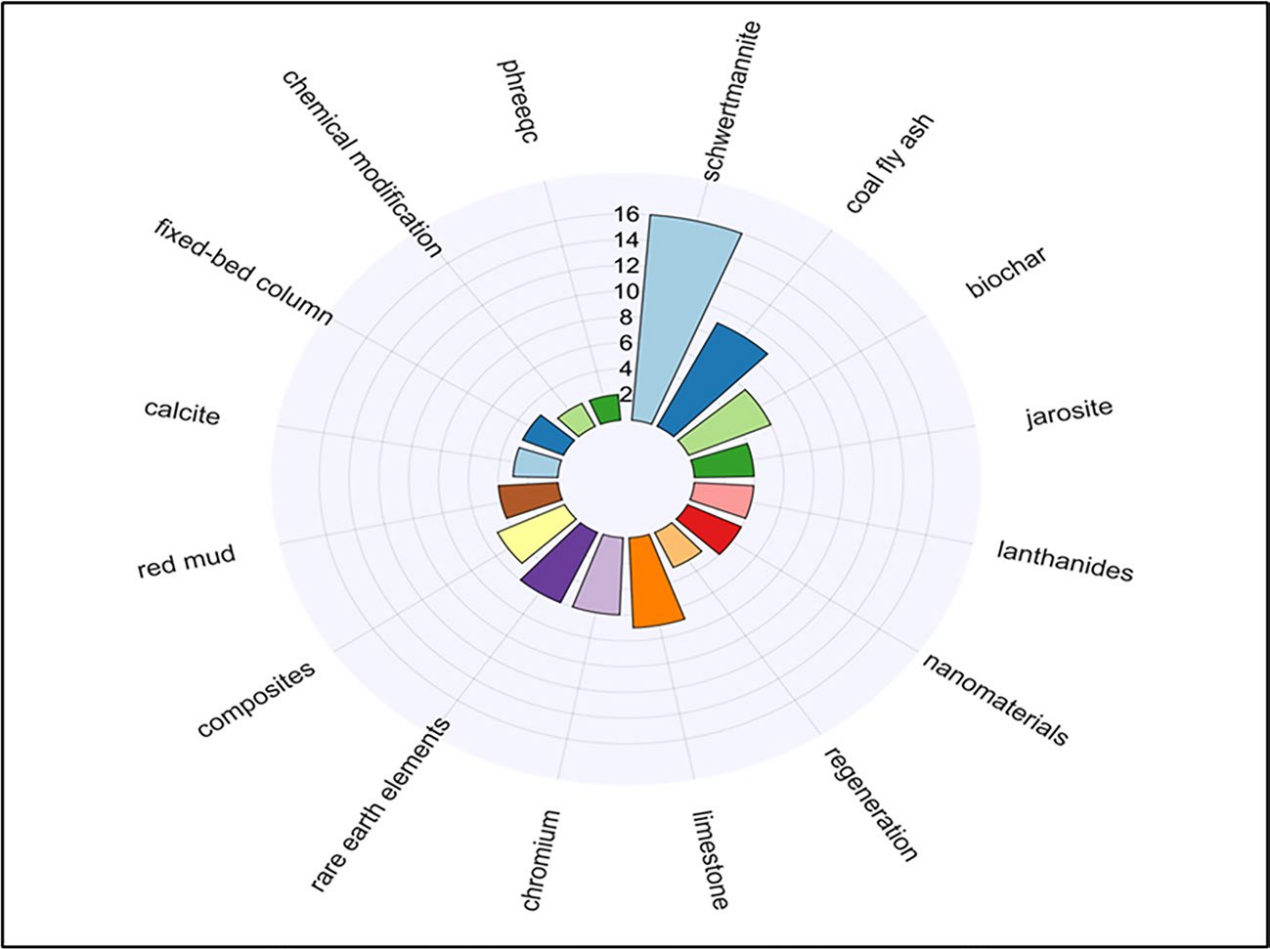
Current research topics associated with adsorption techniques for AMD treatment (2020–2022) (created using Chiplot)

The projected directions for future research and current research trends were identified by analyzing the keywords from authors’ publications from 2020 to 2022. Sixteen topics have emerged as significant trends that are likely to continue to be influential beyond 2022 (Fig. 13).

These critical areas of focus highlight current research trends. Among the identified thematic topics, schwertmannite emerged as the most frequently discussed topic, with a frequency of 16. Other prominently researched areas include coal fly ash (9), limestone (7), biochar (6), rare earth elements (6), composites (5), and nanomaterials (4). New thematic areas such as regeneration and modeling are also emerging and enticing the interest of researchers. These findings highlight the diverse range of materials and concepts explored for effective AMD remediation through adsorption techniques.

Schwertmannite is a promising adsorbent material known for its high adsorption capacity and ability to remove various contaminants from AMD-affected water, particularly for natural attenuation (Carrero et al. 2022). The emergence of thematic topics like biochar, nanomaterials, chemical modifications, and composites signify the exploration of innovative materials in pursuit of broader adsorption capacities for multimetal ionic streams and improved adsorption efficiencies (Sun and Wang 2023). Furthermore, research has shown an increasing preference for natural, abundant, cheap adsorbents, such as coal fly ash for removing heavy metals as an attractive option for sustainable and cost-effective remediation approaches (Kalombe et al. 2020).

The inclusion of limestone and calcites reflects the continued exploration of alkaline materials for the simultaneous decontamination and neutralization of AMD and the feasibility of fixed bed columns as treatment technologies for long-term AMD remediation. Limestone application as a material has remained significant over the years (Rötting et al. 2008; Ding et al. 2023). Their presence in the identified topics indicates an ongoing interest in studying their adsorption properties and optimizing their utilization in AMD remediation strategies. Chromium, lanthanides, and rare earth elements contaminants as thematic topics reflect the interest in addressing specific contaminant removal challenges in AMD treatment (Butu et al. 2023). The research focusing on the adsorptive behavior of these materials demonstrates the efforts to develop tailored adsorbents capable of effectively sequestering these contaminants. Researchers are also committed to exploring modeling tools like PHREEQR to predict and optimize adsorption processes, contributing effectively toward managing AMD pollutants.

Overall, the identified thematic topics illustrate the diverse range of materials, concepts, and contaminants explored in adsorption for AMD treatment. The findings highlight the dynamic nature of research in this field and the ongoing pursuit of innovative and sustainable approaches for mitigating the environmental impact of AMD. The results of this analysis contribute to the current knowledge base, providing researchers with valuable insights and directions for further exploration in the quest for effective AMD remediation using adsorption techniques.

## Conclusions and future prospects

This review used bibliometric analysis to investigate research articles on AMD treatment using adsorption techniques from 2013 to 2022. The study combined Scopus and WoSCC datasets to gather relevant data. The findings showed a steady annual growth rate of about 11% in article production over the specified period. China, the United States, and South Africa were the leading countries in article productivity, while the most productive and influential institutions and authors mainly belonged to these countries. The journal *Environmental Science and Pollution Research* was the most prolific in this field, publishing 25 articles on the topic.

Adsorption has shown its potential as an effective and cost-efficient technique for treating AMD due to its versatility and ability to use a wide range of adsorbents. The recovery of rare earth elements and the reusability of adsorbents play crucial roles in promoting the circular economy. The main clusters in this research topic involved AMD, adsorption, and heavy metal ions. Trend analysis suggests that current and future research will focus on developing novel adsorbents. Researchers are exploring nanomaterials, nanocomposites, clays, coal fly ash, biochar, and composites to efficiently and selectively remove pollutants and recover valuable resources like rare earth elements. Researchers are also investigating using fixed bed columns for concept validation and commercialization. Additionally, studies on adsorbent regeneration or reusability remain of interest, aiming to develop effective strategies for managing spent adsorbents.

The review showed the potential and challenges of adsorption in AMD treatment. It offered valuable insights for future research directions, helping researchers, policymakers, and stakeholders to develop effective and sustainable strategies for AMD remediation using adsorption techniques. In this research field, the main clusters were acid mine drainage, adsorption, and heavy metal ions. Trend analysis showed current research efforts toward finding new adsorbents such as coal fly ash, biochar, nanomaterials, and composites to remove emerging contaminants and recover rare earth metals. Additionally, initial regeneration studies remained a topic of interest in finding clean methods of managing toxic spent adsorbents. Furthermore, researchers explored the use of fixed bed columns, potentially for the validation for commercialization. Overall, this review emphasized the potential of adsorption in AMD treatment and the ongoing need for continued research into novel and effective adsorbents.

In conclusion, addressing the challenges posed by AMD requires a global effort and collaboration among nations, particularly those heavily involved in intensive mining activities. It is imperative to include the participation of countries from various regions and impoverished African nations where regulatory frameworks for assessing the impact of AMD may be lacking. By fostering international cooperation, resources, expertise, and knowledge can be pooled together to tackle this environmental crisis effectively. Furthermore, embracing cutting-edge technologies such as artificial intelligence (AI) and machine learning can significantly enhance our understanding of the complex nature of AMD. These tools offer valuable insights, enabling researchers to develop innovative solutions and preventive measures. By uniting efforts and leveraging advanced technologies, we can pave the way for a sustainable and responsible mining industry that minimizes the environmental impact of AMD, ensuring a better future for generations to come.

## Supplementary Information

Below is the link to the electronic supplementary material.

Supplementary file1 (DOCX 35 KB)

## Data Availability

Upon request, all data and materials utilized in this study will be made readily accessible.
