# Supplementary material for: Exploring acid mine drainage treatment through adsorption: a bibliometric analysis
Source: Environ Sci Pollut Res Int. 2024 Oct 1;31(50):59659–80. doi: 10.1007/s11356-024-35047-2 (PMC11519127; doi:10.1007/s11356-024-35047-2)
Supplement: Supplementary file 1 — Supplementary file1 (DOCX 35 KB) [file 11356_2024_35047_MOESM1_ESM.docx]

**Supplementary Material:**

**Exploring acid mine drainage treatment through adsorption: A bibliometric analysis**

Vuyiswa Dube^a^, Zebron Phiri^a^*, Alex Tawanda Kuvarega^a^, Bhekie Brilliance Mamba^a^, Lueta-Ann de Kock^a^*

*^a^Institute for Nanotechnology and Water Sustainability (iNanoWS), College of Science Engineering and Technology, University of South Africa, Florida Campus, Roodepoort, Gauteng 1709, South Africa*

*Corresponding author:

Email address: [phiriz@unisa.ac.za](mailto:phiriz@unisa.ac.za) (Zebron Phiri)

**Table 1:** Guidelines for exporting and merging of Scopus and Web of Science datasets.

| Purpose | Method |
| --- | --- |
| Export data from Scopus and WoSCC. | **Scopus dataset**: Export selection as a BibTeX file to include information on citations, bibliography, abstract and keywords, and references. Exclude all funding details.  Assume M1=Scopus dataset |
|  | WoSCC dataset: Export selection as a plain text file to include a complete record with cited references. The data size limits the export to 500 journal articles at a time. Export in batches if you have large volumes of data.  M2 = WoSCC dataset for 500 articles in plain text  M3 = WoSCC dataset for 501 - 797 articles. plain.text |
| Load and install the RStudio and bibliometric software packages. | Download the RStudio software.  Input and run the following commands:  >install. packages (“Bibliometrix”)  >library (Bibliometrix)  >Biblioshiny |
| Define the pathway for accessing exported Scopus and WoSCC data from computer files. | Setwd (“C:/users/file name”)  Getwd () |
| Import Scopus dataset. | Input and run the command:  Scopus_data<-convert2df (“M1.bib”, dbsource,” Scopus”, format= “BibTeX”) |
| Combine plain text files from the WoSCC dataset.  (Convert plain text to a BibTeX file format) | Input and run the commands:  M4 <- c(readlines(“M2.txt”) “\n”, headlines (M3.txt”))  M4 <- paste (big_txt, collapse, “\n”)  Writelines (M4.txt)  M4 = combined WoSCC txt files |
| Import WoSCC dataset. | Input and run command:  M5 <- convert2df (“M4.txt”) |
| Merge Scopus and WoSCC datasets.  Remove any existing duplicates. | Input and run command:  M6 <- mergeDbSources (M5, scopus_data, remove. duplicate = T) |
| Convert a merged document to a library format xls file. | Input and run command:  Write.xlsx (M6, “M6.xlsx”) |
| Save in an Excel format. | Input and run command:  Library (openxlsx)  Write.xlsx (M6, file = “Merged dataset”) |
| Assess the Excel file. | Remove remaining duplicates using the Microsoft Excel command “remove duplicates.”  Eliminate any irrelevant articles. |
| Open Biblioshiny to analyze merged datasets. | Input and run command:  Biblioshiny () |
